# Two new species and five newly recorded species of the genus *Udea* Guenée from China (Lepidoptera, Crambidae)

**DOI:** 10.3897/zookeys.565.6304

**Published:** 2016-02-17

**Authors:** Dandan Zhang, Jinwei Li

**Affiliations:** 1 Institute of Entomology/State Key Laboratory of Biocontrol/The Museum of Biology, Sun Yat-sen University, Guangzhou, Guangdong 510275, China

**Keywords:** Lepidoptera, Crambidae, *Udea*, checklist, taxonomy, China

## Abstract

A checklist of the 31 Chinese species of *Udea* is given, including the new species and new records. *Udea curvata* **sp. n**. and *Udea albostriata* **sp. n**. are described and illustrated. *Udea exigualis* (Wileman, 1911), *Udea stationalis* Yamanaka, 1988, *Udea prunalis* (Denis & Schiffermüller, 1775), *Udea elutalis* (Denis & Schiffermüller, 1775) and *Udea cyanalis* (La Harpe, 1855) are newly recorded for China.

## Introduction

*Udea* Guenée is a large genus, with more than 210 species, and is mainly distributed in the temperate Eurasia and in the New World, with a remarkable number of endemic species occuring on islands in the Pacific and Atlantic Oceans, on the Hawaiian Islands and some other islands (Nuss et al. 2003–2014; Mally and Nuss 2011; Slamka 2013). *Udea* is usually placed in the Spilomelinae (Munroe 1995; Solis and Maes 2003), but this placement is not confirmed by phylogenetic study (Mally and Nuss 2011).

Morphology and genitalia of *Udea* are simple and uniform throughout the genus. Species of *Udea* are dark to light greyish, brown, reddish-yellow, dark yellowish or pale yellow; the forewing has a circular and a reniform cellular stigmata; the hindwing bears a streak at the anterior angle and a dot at the posterior angle of cell; the wings usually with marginal dots at ends of veins. Male genitalia with uncus inverted T-shaped, apex bulbous, with setae; fibula extending ventrad to distad. Corpus bursae usually with a large lanceolate, denticulate signum in female genitalia.

Important taxonomic contributions on *Udea* were published by Munroe (1950, 1966, 1989, 1995), Zimmerman (1958), Hannemann (1964), Inoue (1982), Yamanaka (1988), Inoue et al. (2008), Mally and Nuss (2011) and Slamka (2013). Chinese species of *Udea* were reported by Walker (1859), Hampson (1899, 1918), Sauber (1899), Leech and South (1901), Zerny (1914), Strand (1918), Caradja (1916, 1925, 1927, 1928), Caradja and Meyrick (1937) and Yamanaka (1972). Two new species and five newly recorded species for China are presented in this study, bringing the total to 31 species recorded in China.

## Material and methods

This study is based on the examination of specimens collected by using light traps. Terminology of the genitalia follows Maes (1995), Mally and Nuss (2011) and Slamka (2013). Genitalia dissection and mounting methods follow Robinson (1976) and Li and Zheng (1996), with some modification. The specimens are deposited in the Biology Museum, Sun Yat-sen University (SYSBM) except those specified with the Insect Collection, College of Life Sciences, Nankai University (NKUM).

## Results

### Checklist of Chinese *Udea* species

***Udea* Guenée, 1845**

*Udea* Guenée in Duponchel, 1845: 209. Type species: *Pyralis ferrugalis* Hübner, 1796, by monotypy.

*Udea albostriata* **sp. n.**

Distribution. China (Hebei).

*Udea aksualis* (Caradja, 1928) (as *Pionea*)

Distribution. China (Xinjiang).

*Udea austriacalis* (Herrich-Schäffer, 1851) (as *Botys*)

*Scopula donzelalis* Guenée, 1854

*Botys sororialis* Heyden, 1860

*Botys nitidalis* Heinemann, 1865

*Pyrausta austriacalis altaica* Zerny, 1914

*Pyrausta austriacalis juldusalis* Zerny, 1914

Distribution. China (Xinjiang), Russia (Altai), France, Switzerland, Austria, Romania, Bulgaria, Albania.

*Udea conubialis* Yamanaka, 1972

Distribution. China (Taiwan).

*Udea costalis* (Eversmann, 1852) (as *Botys*)

*Botys hilaralis* Christoph, 1881

Botys hyperborealis var. hoffmanni Krulikovsky, 1898

Pionea costalis var. alaicalis Caradja, 1916

Pionea costalis var. alaicalis f. brunnealis Caradja, 1916

*Mesographe itysalis maurinalis* Curtis, 1934

Distribution. China (Xinjiang), Mongolia, Russia (Far East, Siberia, Altai), France, Lithuania, Poland.

*Udea curvata* **sp. n.**

Distribution. China (Tibet).

*Udea cyanalis* (La Harpe, 1855) (as *Botys*), **new record to China**

Distribution. China (Hebei), Spain, France, Herzegovina, Romania, Germany, Central Urals, Russia (Caucasus).

*Udea decrepitalis* (Herrich-Schäffer, 1848) (as *Botys*)

Pionea decrepitalis ab. leucoalis Strand, 1920

Distribution. China (Hebei, Qinghai) (Xu, 1997), Europe.

*Udea defectalis* (Sauber, 1899) (as *Botys*)

Distribution. China (Qinghai).

*Udea elutalis* (Denis & Schiffermüller, 1775) (as *Pyralis*), **new record to China**

*Pyralis albidalis* Hübner, 1796

Distribution. China (Hebei, Xinjiang), Kazakhstan, West Europe, Central Europe, Romania, Balticum, Finland, Russia (Siberia).

*Udea endotrichialis* (Hampson, 1918) (as *Hapalia*)

Distribution. China (Taiwan).

*Udea exigualis* (Wileman, 1911) (as *Pionea*), **new record to China**

Distribution. China (Fujian, Guangxi, Guizhou, Hubei, Hunan, Sichuan, Tibet, Yunnan), Japan.

*Udea ferrugalis* (Hübner, 1796) (as *Pyralis*)

*Scopula martialis* Guenée, 1854

*Scopula hypatialis* Walker, 1859

*Pionea maculata* Costantini, 1923

*Pionea obsoleta* Costantini, 1923

*Pionea granjalis* Chrétien, 1925

Udea martialis f. fusca Dufrane, 1960

Udea martialis f. pallida Dufrane, 1960

Distribution. China (Gansu, Guangdong, Guizhou, Hebei, Henan, Hubei, Hunan, Jiangsu, Qinghai, Shaanxi, Shandong, Shanxi, Shanghai, Sichuan, Taiwan, Tianjin, Yunnan, Zhejiang), widely distributed in Asia, Europe and Africa.

*Udea flavofimbriata* (Moore, 1888) (as *Mabra*)

*Botys obealis* Snellen, 1899

Distribution. China (Guangdong, Taiwan), Japan, Myanmar, Indonesia (Sumatra, Java), India, Sri Lanka.

*Udea fulcrialis* (Sauber, 1899) (as *Botys*)

Distribution. China (Qinghai).

*Udea incertalis* (Caradja in Caradja & Meyrick, 1937) (as *Pionea*)

Distribution. China (Yunnan).

*Udea lugubralis* (Leech, 1889) (as *Botys*)

Distribution. China (Fujian, Guizhou, Henan, Hubei, Hunan, Shaanxi, Sichuan, Yunnan, Zhejiang), Korea, Japan, Russia (Sakhalin, Shikotan Island, Ussuri, Amur).

*Udea nigrostigmalis* Warren, 1896

Distribution. China (Guangdong), India.

*Udea montensis* Mutuura, 1954

Distribution. China (Hubei, Sichuan) (Song, 2001), Japan.

*Udea orbicentralis* (Christoph, 1881) (as *Botys*)

Distribution. Western China, Korea, Japan, Russia (Vladivostok).

*Udea planalis* (South in Leech & South, 1901) (as *Pionea*)

Distribution. China (Sichuan).

*Udea poliostolalis* (Hampson, 1918) (as *Hapalia*)

Distribution. China (Taiwan).

*Udea prunalis* (Denis & Schiffermüller, 1775) (as *Pyralis*), **new record to China**

*Phalaena nivealis* Fabricius, 1781

*PhalaenaPyralis ferruginalis* Villers, 1789

*Pyralis leucophaealis* Hübner, 1796

*Pyralis nebulalis* Haworth, 1811

Distribution. China (Gansu, Heilongjiang, Ningxia, Shanxi, Sichuan, Xinjiang), Europe (except some of Mediterranean Islands).

*Udea russispersalis* (Zerny, 1914) (as *Pionea*)

Distribution. China (Xinjiang).

*Udea schaeferi* (Caradja in Caradja & Meyrick, 1937) (as *Pionea*)

Distribution. China (Yunnan).

*Udea scoparialis* (Hampson, 1899) (as *Pionea*)

Distribution. China (Tibet).

*Udea stationalis* Yamanaka, 1988, **new record to China**

Distribution. China (Fujian), Japan.

*Udea subplanalis* (Caradja in Caradja & Meyrick, 1937) (as *Pionea*)

Distribution. China (Yunnan).

*Udea suisharyonensis* (Strand, 1918) (as *Pionea*)

*Pionea lolotialis* Caradja, 1927

Distribution. China (Sichuan, Taiwan).

*Udea thyalis* (Walker, 1859) (as *Botys*)

Distribution. China, Japan.

*Udea tritalis* (Christoph, 1881) (as *Botys*)

Distribution. Northern China, Korea, Japan, Russia (Ussuri) (Inoue, 1993).

### Descriptions of new species and diagnoses of new records to China

#### Udea exigualis

Taxon classificationAnimaliaLepidopteraCrambidae

(Wileman, 1911) new record to China

Pionea exigualis Wileman, 1911: 388. Type locality: Japan.
Udea exigualis (Wileman): Inoue 1982: 364.

##### Diagnosis.

This species is similar to other species of *Udea lugubralis*-complex. It can be distinguished from *Udea lugubralis* by smaller size (wingspan 16–21 mm) and longer harpe with sharp point. It differs from *Udea stationalis* and *Udea montensis* by bent harpe with sharp point. Its phallus apodeme lacking a small lateral tooth-like process is different from *Udea montensis*. *Udea exigualis* is similar to *Udea ferrugalis* and *Udea testacea* (Butler) with yellowish-brown forewing bearing dark brown fringe, but can be distinguished in male genitalia by the more slender and shorter fibula and the juxta without dorsal arms.

##### Material examined.

**China: Fujian**: 1♂, Yong’anyan, Mt. Daiyunshan, 1300 m, 12-IX-2002, coll. Xinpu Wang (NKUM); 1♀, Guadun, Mt. Wuyishan, 27°74'N, 117°64'E, 1220 m, 18-V-2012, coll. Jinwei Li, genitalia slide no. LJW12156; **Guangxi**: 1♂, Gaozhai, Xing’an, 28-VIII-2011, coll. Jinwei Li, genitalia slide no. LJW12253; 7♂, Anjiangping Reserve, 25°33'N, 109°55'E, 1751 m, 10-VII-2013, coll. Xiaohua Chen, genitalia slide no. LJW12207; 1♂, Jiuniutang, Mt. Maoershan, 550 m, 20-IV-2002, coll. Shulian Hao, Huaijun Xue (NKUM); **Guizhou**: 4♂3♀, Mt. Leigongshan, 26°21'N, 108°09'E, 1198 m, 14–15-VII-2013, coll. Xiaohua Chen, genitalia slides no. LJW12255 (♀), LJW12269 (♀), LJW12270 (♂); 4♂7♀, Huguosi, Mt. Fanjingshan, 1300 m, 1–3-VIII-2001, coll. Houhun Li, Xinpu Wang (NKUM); 1♂, Jinding, Mt. Fanjingshan, 2100 m, 31-VII-2001, coll. Houhun Li, Xinpu Wang (NKUM); 1♀, Huixiangping, Mt. Fanjingshan, 1700 m, 1-VI-2002, coll. Xinpu Wang (NKUM); 1♂, Suoluo, Chishui, 390 m, 30-V-2000, coll. Yanli Du (NKUM); **Hubei**: 1♂, Jiuhuping, Shennongjia, 31°30'N, 110°21'E, 1888 m, 9-IX-2012, coll. Lijun Yang; 1♂, Muyu, Shennongjia, 31°28'N, 110°23'E, 1072 m, 8-IX-2012, coll. Jinwei Li, genitalia slide no. LJW12150; 1♀, Maoping, Wufeng, 30°08'N, 110°40'E, 1175 m, 12-IX-2012, coll. Lijun Yang, genitalia slide no. LJW12263; 6♂5♀, Shayuan, Hefeng, 1260 m, 15–18-VII-1999, coll. Houhun Li (NKUM); 1♂, Houhe, Wufeng, 1100 m, 11-VII-1999, coll. Houhun Li (NKUM); 5♂, Pingbaying, Xianfeng, 1280 m, 21–22-VII-1999, coll. Houhun Li (NKUM); 2♂, Maoba, Lichuan, 700 m, 30-VII-1999, coll. Houhun Li (NKUM); **Hunan**: 4♂3♀, Mt. Badagongshan, Sangzhi, 1250 m, 12-VIII-2001, coll. Houhun Li, Xinpu Wang (NKUM); 3♂, Zhangjiajie, 650 m, 7–11-VIII-2001, coll. Houhun Li, Xinpu Wang (NKUM); **Sichuan**: 1♂, Labahe, Tianquan, 30°09'N, 102°26'E, 1860 m, 8-VII-2012, coll. Jinwei Li, genitalia slide no. LJW12250; **Tibet**: 1♂, Dexing, Motuo, 29°20'N, 95°18'E, 835 m, 9-VII-2013, coll. Jinwei Li, genitalia slide no. LJW12209; 1♂, Pailong, Linzhi, 30°01'N, 95°00'E, 2010 m, 5-VII-2013, coll. Jinwei Li, genitalia slide no. LJW12212; **Yunnan**: 1♂1♀, Baihualing, Baoshan, 1520 m, 11–13-VIII-2007, coll. Dandan Zhang, genitalia slide no. LJW12160 (♂); 1♂, Haba, Diqing, 15-VII-2011, coll. Jinwei Li, genitalia slide no. LJW12153.

##### Distribution.

China (Fujian, Guangxi, Guizhou, Hubei, Hunan, Sichuan, Tibet, Yunnan), Japan.

#### Udea stationalis

Taxon classificationAnimaliaLepidopteraCrambidae

Yamanaka, 1988 new record to China

Udea stationalis Yamanaka, 1988: 111. Type locality: Japan, Honshu.

##### Diagnosis.

This species is similar to other species of *Udea lugubralis*-complex. It can be distinguished from *Udea lugubralis* by smaller size (wingspan 15–20 mm). Differs from both *Udea lugubralis* and *Udea exigualis* by somewhat straight harpe, by lacking granularly membranous interval zone between antrum and colliculum. Differs from *Udea montensis* by the phallus apodeme lacking the small lateral tooth-like process, and by lacking granularly membranous interval zone between antrum and colliculum.

##### Material examined.

**China: Fujian**: 1♀, Guadun, Mt. Wuyishan, 27°74'N, 117°64'E, 1220 m, 18-V-2012, coll. Jinwei Li, genitalia slide no. LJW12154.

##### Distribution.

China (Fujian), Japan.

#### Udea curvata sp. n.

Taxon classificationAnimaliaLepidopteraCrambidae

http://zoobank.org/0E665363-306D-4736-AB9B-47CF4B8E5869

Figs 1 , 4

##### Type-locality.

China, Tibet, Milin, Paizhen, 29°30'N, 94°51'E, 2961 m, 2-VII-2013, coll. Jinwei Li.

##### Type material.

**Male holotype**, **China: Tibet**: Paizhen, Milin, 29°30'N, 94°51'E, 2961 m, 2-VII-2013, coll. Jinwei Li, genitalia slide no. LJW12172 (SYSBM); **Paratypes.** 3♂, **China: Tibet**: Paizhen, Milin, 29°30'N, 94°51'E, 2961 m, 2–3-VII-2013, coll. Jinwei Li, genitalia slides no. LJW12248, LJW12267 (SYSBM). **Additional material.** 1 abdomen missing, **China: Tibet**: Paizhen, Milin, 29°30'N, 94°51'E, 2961 m, 2-VII-2013, coll. Jinwei Li.

##### Diagnosis.

This species is similar to *Udea decrepitalis* and *Udea elutalis* with zigzaggy serrated postmedian line and darker postmedian area of forewing, but can be distinguished in: fibula claw-shaped, bent, with point apex; phallus with a thumb-shaped cornutus. Differs from *Udea decrepitalis* also by colouration of forewing stigmata identical with ground colour. *Udea curvata* is similar to *Udea conubialis* in male genitalia, but can be distinguished in: wingspan 25.5–28.5 mm, ground colour yellow, postmedian line zigzaggy, proximal cellular stigma distinct, fibula strongly bent. *Udea curvata* similar to *Udea lutealis* with yellow ground colour and colouration of forewing stigmata identical with ground colour, but can be distinguished by bent fibula extending ventro-distally, juxta bifid ventrally, phallus with a thumb-shaped cornutus, posterior phallus with granulated area not sclerotised, and lacking projecting denticulate ridge most posteriorly.

##### Description.

**Figures 1–3.**
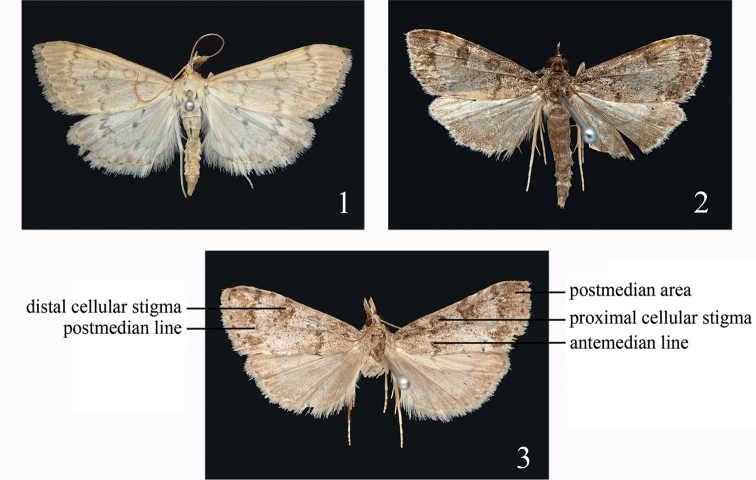
Adults of *Udea* species. **1** *Udea curvata* sp. n., male, paratype, Paizhen **2–3** *Udea albostriata* sp. n. **2** paratype, male, Taomugeda **3** paratype, female, Taomugeda.

Male (Fig. 1). Wingspan 25.5–28.5 mm. Frons yellowish-brown, with white lateral band not extending to anterior end, and a faint, short middle band. Vertex pale yellowish-brown. Labial palpus slightly upturned obliquely, third segment porrect; length about 2.5 times diameter of eye; yellowish-brown, contrastingly white at base ventrally. Maxillary palpus yellowish-brown, with a brush of scales. Basal scaling of proboscis white. Antenna with yellowish-brown scales dorsally. Thorax and abdomen yellow dorsally, dirty white ventrally. Legs creamy white, foreleg inner side dark yellowish.

Forewing yellow, scattered with brown scales, markings grey-brown; antemedian line from costal 1/4 sinuated to 1/3 posterior margin; proximal cellular stigma circular; distal cellular stigma kidney-shaped; postmedian line zigzaggy serrate, from costal 3/4, excurved around cell, and strongly inflexed below distal cellular stigma, then to 2/3 on posterior margin; postmedian area strongly dusted with grey and alternately formed grey and yellow streaks; vein ends on wing margin each with a small brown dot; fringe yellow, basal 1/4 grey. Hindwing pale yellow, a darker steak at anterior angle and a blackish dot at posterior angle of cell; postmedial line grey-brown, zigzaggy serrate, with anterior 1/4 most distinct; postmedian area similar to forewing, marginal line and fringe as forewing, paler at tornus area.

**Figures 4–11.**
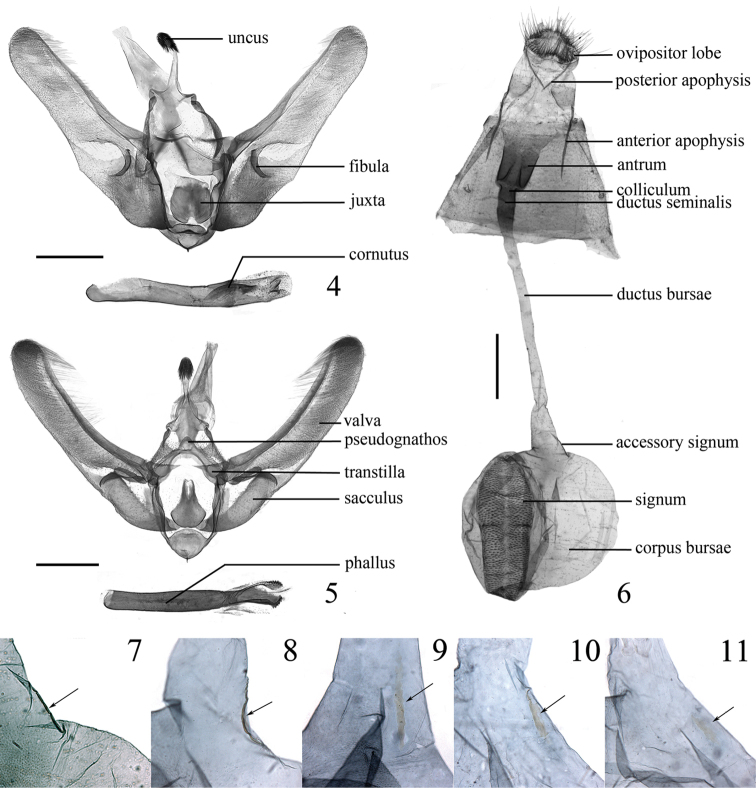
Genitalia of *Udea* species. **4** Male genitalia of *Udea curvata* sp. n., genitalia slide no. LJW12267 **5–11** *Udea albostriata* sp. n. **5** Male genitalia, genitalia slide no. LJW12288 **6** Female genitalia, genitalia slide no. LJW12296 **7–11** Variation of accessory signum, genitalia slides no. LJW12296, LJW12297, LJW12292, LJW12284, LJW12287. Scale bars: 0.5 mm.

**Male genitalia** (Fig. 4). Uncus inverted T-shaped, with base expanded, apex bulbous and setose dorso-laterally. Pseudognathos slender and ribbon-like, semicircular produced medially. Triangular transtilla connected. Valva narrow and long, costa nearly straight, proximal half of costa twice as broad as distal half, ventral margin broadly sinuate basally, with a stout tip protruding proximal of the distal end of sacculus, nearly parallel to costa from middle to end; fibula claw-shaped, bent ventro-distally, with point apex. Saccus inflated, ventrally keeled. Juxta nearly circular, somewhat bifid ventrally, dorsal edge serrated. Phallus cylindrical, with a short coecum, with a thumb-shaped cornutus, posterior phallus with granulated vesica.

Female unknown.

##### Distribution.

China (Tibet).

##### Etymology.

The specific name is derived from the Latin *curvata* = curved, referring to the curved fibula.

#### Udea prunalis

Taxon classificationAnimaliaLepidopteraCrambidae

(Denis & Schiffermüller, 1775) new record to China

Pyralis prunalis Denis & Schiffermüller, 1775: 121. Type locality: Austria, Vienna environs.
Phalaena nivealis Fabricius, 1781: 274.
Phalaena Pyralis ferruginalis Villers, 1789: 451.
Pyralis leucophaealis Hübner, 1796: 27.
Pyralis nebulalis Haworth, 1811: 386.
Pionea prunalis (Denis & Schiffermüller): Hampson 1899: 243.
Udea prunalis (Denis & Schiffermüller): Hasenfuss 1960: 182.

##### Diagnosis.

*Udea prunalis* is similar to *Udea cyanalis*, *Udea inquinatalis* (Lienig & Zeller), *Udea orbicentralis*-complex and *Udea albostriata* sp. n. with greyish white ground colour of forewing variably dusted with dark brown, proximal cellular stigma, distal cellular stigma and postmedian area strongly and contrastingly dark browned, but can be distinguished from them in: cornuti composed of a row of linked short spines, a row of closely squeezed long spines and a single longer spine in male genitalia, the mid-folded ductus bursae with posterior half sclerotised and plate-shaped accessory signum in female genitalia.

##### Material examined.

**China: Gansu**: 5♂3♀, Mt. Xinglongshan, Yuzhong, 2120–2230 m, 29-VII–4-VIII-1993, coll. Houhun Li (NKUM); **Heilongjiang**: 1♂, Jiagedaqi, 14-VII-2012, coll. Dandan Zhang, Lijun Yang, genitalia slide no. LJW12157 (♂); **Ningxia**: 1♂, Xinmin Forestry Station, Jingyuan, 2100 m, 7-VIII-2000, coll. Houhun Li, Shuxia Wang (NKUM); **Shanxi**: 1♀, Xiachuan, Qinshui, 35°26'N, 112°00'E, 1555 m, 24-VII-2013, coll. Weicai Xie; **Sichuan**: 10♂6♀, Rize, Jiuzhaigou, 2700 m, 13-VIII-2002, coll. Shulian Hao (NKUM); 1♂, Shuzheng, Jiuzhaigou, 2300 m, 17-VIII-2002, coll. Shulian Hao (NKUM); 11♂3♀, Zhawa, Jiuzhaigou, 2400 m, 15-VIII-2002, coll. Shulian Hao (NKUM); **Xinjiang**: 1♂, Kuerdening, Gongliu, 2230 m, 28-VII-1994, coll. Houhun Li, Hongyan Qin (NKUM); 1♀, Kuerdening, Gongliu, 43°10'N, 82°52'E, 1483 m, 22-VII-2013, coll. Jinwei Li, genitalia slide no. LJW12254.

##### Distribution.

China (Gansu, Heilongjiang, Ningxia, Shanxi, Sichuan, Xinjiang), Europe (except some Mediterranean Islands).

#### Udea elutalis

Taxon classificationAnimaliaLepidopteraCrambidae

(Denis & Schiffermüller, 1775) new record to China

Pyralis elutalis Denis & Schiffermüller, 1775: 121. Type locality: Austria, Vienna environs.
Pyralis albidalis Hübner, 1796: fig. 118.
Udea elutalis (Denis & Schiffermüller): Hasenfuss 1960: 182.

##### Diagnosis.

This species is similar to *Udea lutealis* (Hübner), but can be distinguished by a wide, blade-shaped fibula with a minute, hook-like apex, by praephallus with cornuti a tight line of spines in male genitalia. *Udea elutalis* with antrum narrower than colliculum in female genitalia but contrary in *Udea lutealis*.

##### Material examined.

**China: Hebei**: 32♂13♀, Taomugeda, Laiyuan County, 39°37'N, 114°59'E, 1420 m, 3-VIII-2013, coll. Weicai Xie, Xiaolin Liu, genitalia slides no. LJW12174 (♂), LJW12203 (♀), LJW12243 (♂), LJW12244 (♀), LJW12268 (♂), LJW12289 (♀), LJW12290 (♂), LJW12291 (♀); **Xinjiang**: 1♂, Tianchi, Fukang, 43°52'N, 88°09'E, 2009 m, 18-VII-2013, coll. Jinwei Li, genitalia slide no. LJW12181; 4♀, Baiyanggou, Nanshan, 43°27'N, 87°11'E, 1947 m, 17-VII-2013, coll. Jinwei Li, genitalia slides no. LJW12202, LJW12294.

##### Remarks.

There is considerable variation in size of wingspan, ground colour and genitalia. The specimens from Hebei have whitish or whitish-grey forewing, with small wingspan size (18–22 mm). The specimens from Hebei and Xinjiang exhibit a slightly curved and shorter row of spines in the posterior phallus compared to material from Europe and Russia (Bolshakov, 2002; Slamka, 2013). In the female genitalia, the accessory signum varies from crescent- or stick-shaped over gradual reduction to complete absence.

##### Distribution.

China (Hebei, Xinjiang), Kazakhstan, West Europe, Central Europe, Romania, Balticstates, Finland, Russia (Siberia).

#### Udea cyanalis

Taxon classificationAnimaliaLepidopteraCrambidae

(La Harpe, 1855) new record to China

Botys cyanalis La Harpe, 1855: 30. Type locality: Europe.
Udea cyanalis (La Harpe): Hannemann 1964: 322.

##### Diagnosis.

*Udea cyanalis* is similar to *Udea prunalis*, *Udea inquinatalis*, *Udea orbicentralis*-complex and *Udea albostriata* sp. n. with similar ground colour and maculation as mentioned in diagnosis of *Udea prunalis*, but can be distinguished from them by the semicircular produced process of pseudognathos with nipple-shaped end in male genitalia. In female genitalia, this species differs from *Udea prunalis*, *Udea inquinatalis* and *Udea grisealis* Inoue, Yamanaka & Sasaki by ductus bursae approximately 1.8 times the length of the corpus bursae, the corpus bursae with narrowly crescent-shaped accessory signum, but lacking the lanceolate signum; differs from *Udea nebulatalis* Inoue, Yamanaka & Sasaki by ductus bursae approximately 1.8 times the length of the corpus bursae and nearly round corpus bursae; differs from *Udea proximalis* Inoue, Yamanaka & Sasaki and *Udea intermedia* Inoue, Yamanaka & Sasaki by crescent-shaped accessory signum but lacking the lanceolate or pyriform signum; differs from *Udea orbicentralis* and *Udea albostriata* sp. n. by lacking the lanceolate signum.

##### Material examined.

**China: Hebei**: 2♂, Taomugeda, Laiyuan County, 39°37'N, 114°59'E, 1420 m, 3-VIII-2013, coll. Xiaolin Liu, genitalia slides no. LJW12282, LJW12293.

##### Distribution.

China (Hebei), Spain, France, Herzegovina, Romania, Germany, Central Urals, Russia (Caucasus).

#### Udea albostriata sp. n.

Taxon classificationAnimaliaLepidopteraCrambidae

http://zoobank.org/B4A2764A-7681-411A-AEFA-BFD4686B8BBE

Figs 2 , 3 , 5–11

##### Type-locality.

China, Hebei, Laiyuan County, Taomugeda, 39°37'N, 114°59'E, 1420 m, 3-VIII-2013, coll. Xiaolin Liu.

##### Type material.

**Male holotype**, **China: Hebei**: Taomugeda, Laiyuan County, 39°37'N, 114°59'E, 1420 m, 3-VIII-2013, coll. Xiaolin Liu, genitalia slide no. LJW12204 (SYSBM); **Paratypes.**14♂8♀, same data as holotype, genitalia slides no. LJW12173 (♂), LJW12178 (♂), LJW12245 (♂), LJW12283 (♂), LJW12284 (♀), LJW12286 (♀), LJW12287 (♀), LJW12288 (♂), LJW12292 (♀), LJW12296 (♀) (SYSBM); 2♀, **Hebei**: Jinhekou, Wei County, 39°57'N, 114°56'E, 1112 m, 5-VIII-2013, coll. Weicai Xie, Xiaolin Liu, genitalia slide no. LJW12297 (♀) (SYSBM). **Additional material. China: Hebei**: 1 abdomen missing, Taomugeda, Laiyuan County, 39°37'N, 114°59'E, 1420 m, 3-VIII-2013, coll. Xiaolin Liu; 1 abdomen missing, Jinhekou, Wei County, 39°57'N, 114°56'E, 1112 m, 5-VIII-2013, coll. Weicai Xie, Xiaolin Liu.

##### Diagnosis.

*Udea albostriata* is closely related to *Udea cyanalis*, *Udea prunalis*, *Udea inquinatalis*, *Udea orbicentralis*-complex with similar ground colour and maculation as mentioned in diagnosis of *Udea prunalis*, but can be distinguished from *Udea cyanalis*, *Udea nebulatalis*, *Udea proximalis* by corpus bursae with a lanceolate signum in female genitalia; differs from *Udea prunalis*, *Udea inquinatalis*, *Udea grisealis* and *Udea intermedia* by long ductus bursae about twice the length of the corpus bursae; differs from *Udea orbicentralis* in: praephallus with a sclerotized, granulated area and a projecting ridge strongly denticulate, antrum much broader and shorter than in *Udea orbicentralis*, and not bulged laterally.

##### Description.

Wingspan 17–23 mm. Frons and vertex dark brown, dusted with light grey. Labial palpus slightly upturned obliquely, third segment porrect, dark brown, dusted with light grey, contrastingly white at base ventrally, length approximate three times the diameter of the eye. Maxillary palpus dark brown, dusted with light grey, with tip a brush of scales. Basal scaling of proboscis creamy white. Antenna with dark scales dorsally. Thorax dark greyish, dusted with light grey dorsally, greyish-white ventrally. Abdomen grey to dark greyish dorsally, greyish-white ventrally. Legs greyish-white, with scattered few dark scales, sometimes mid- and hind-tibiae, tarsus dark brown, dusted with white outwardly.

Forewing ground colour greyish white, dusted with dark brown, proximal and distal cellular stigmata and postmedian area strongly and contrastingly dark browned; antemedian line from 1/5 of costa oblique outwards to posterior margin of cell, then sinuating to 1/3 of posterior margin; proximal cellular stigma transversely oval, dark brown, rimmed with blackish; distal cellular stigma nearly 8-shaped, coloured like proximal cellular stigma; postmedian line sinuate, from costal 4/5 slightly arched to 3/5 of CuA_2_, followed by a V-shaped curve, then to 2/3 of posterior margin, traced by a greyish-white line in postmedian area; marginal brown dots at vein ends on costa and termen; basal half of fringe pale grey, distal half dirty white. Hindwing grey, markings indistinct; a dark steak at anterior angle and a blackish dot at posterior angle of cell; postmedian line very indistinct, parallel with termen; fringe paler than in forewing.

**Male genitalia** (Fig. 5). Uncus with basal half nearly triangular, apex bulbous and setose dorso-laterally. Pseudognathos slender and ribbon-like, roundly triangular medially. Transtilla triangular. Valva narrow and elongate, costa slightly concave, slightly tapering in thickness towards apex, nearly parallel-sided with ventral valva edge; fibula extending ventrad, weakly sclerotised, blade-shaped, curved, with tip pointed; sacculus slightly inflated. Saccus ventrally keeled. Juxta broad ventrally, tapered dorsally, with dorsal 1/3 bifid. Phallus cylindrical, slightly curved, with posterior phallus apodeme divided into a sclerotised, granulated area and a projecting denticulate ridge most posteriorly.

**Female genitalia** (Figs 6–11). Ovipositor lobes flat, crescent-shaped, densely setose. Anterior apophyses a little longer than posterior apophyses. Antrum sclerotised, nearly cylindrical, slightly tapering anteriorly, mesoventrally with two longitudinal ridges. Ductus bursae slender, about twice the length of the corpus bursae, slightly sclerotised posteriorly, colliculum short, ductus seminalis from ductus bursae close to colliculum. Corpus bursae nearly round, accessory signum (Figs 7–11) narrowly crescent-shaped, or weakly rod-shaped; signum lanceolate, ends rounded, with a mesally interrupted transverse ridge in the middle.

##### Distribution.

China (Hebei).

##### Etymology.

The specific name is derived from the Latin *albus* = white, *striatus* = striate, referring to forewing postmedian line traced by a greyish-white line in the postmedian area.

## Discussion

*Udea* is one of the most species-rich genera of Spilomelinae. Until now, 31 *Udea*-species are recorded from China, but our knowledge about this fauna is still poor. For example, some of the species are only known by their original descriptions, based on type-localities in China.

Biogeographically, the northern part of China belongs to the Palaearctic region and the southern part to the Oriental region. The border is given by the Qinling Mountains and Huaihe River (Zheng and Zhang 1956). Accordingly, 15 of the Chinese *Udea*-species belong to the Palaearctic fauna, nine to the Oriental fauna and seven occur in both of these or even more regions. Most of the Oriental species occur in the mountains. Therefore, *Udea* could be called a group of temperate regions as well as of mountain regions at more southerly latitudes.

Remarkably, 15 of the *Udea* species recorded from China are so far only known from China. They are distributed in southwestern Yunnan and Sichuan, northwestern Qinghai, Tibet and Xinjiang as well as on Taiwan. Understanding this pattern will require further faunistic investigations throughout China, and a phylogeographic analysis including areas outside China.

## Supplementary Material

XML Treatment for Udea exigualis

XML Treatment for Udea stationalis

XML Treatment for Udea curvata

XML Treatment for Udea prunalis

XML Treatment for Udea elutalis

XML Treatment for Udea cyanalis

XML Treatment for Udea albostriata
